# Multimorbidity is associated with TV-viewing, but not with other types of screen-based behaviors in Brazilian adults

**DOI:** 10.1186/s12889-022-14365-5

**Published:** 2022-10-31

**Authors:** Luciana L. S. Barboza, André O Werneck, Raphael H O Araujo, Luiz G G Porto, Danilo R Silva

**Affiliations:** 1 Study Group in Physiology and Epidemiology of Exercise and Physical Activity (GEAFS), Postgraduate Program in Physical Education, University of Brasília (UnB), Brasília, DF Brazil; 2 Center for Epidemiological Research in Nutrition and Health, Department of Nutrition, School of Public Health, Universidade de São Paulo (USP), São Paulo, SP Brazil; 3 Graduation Program in Health Sciences, Londrina State University (UEL), Londrina, PR Brazil; 4 Faculty of Health Sciences, Universidad Autónoma de Chile, Providencia, Chile; 5 Postgraduate Program in Physical Education, University of Brasília, Campos Darcy Ribeiro, 70910-900 Brasília, Distrito Federal Brazil; 6 Department of Sports and Computer Science, Universidad Pablo de Olavide (UPO), 41013 Seville, Spain

**Keywords:** Chronic disease, Epidemiology, Health behavior, Morbidity, Public health

## Abstract

**Background:**

The presence of multimorbidity increases the risk of mortality, and identifying correlates of multimorbidity can direct interventions by targeting specific modifiable correlates. Here we aimed to investigate the association between two types of screen-based behaviors and multimorbidity.

**Methods:**

We used data from 87,678 Brazilian adults from the National Health Survey (2019). Multimorbidity (presence of two or more chronic conditions among 12 possibilities), TV-viewing, and time on other types of screens (computer, tablet, or cell phone), were self-reported. Crude and adjusted binary and multinominal logistic regression models were performed stratified by sex, age group, and the number of chronic conditions.

**Results:**

Considering adjusted values, 2 h/day as a reference, and reporting values in odds ratio (OR) and prevalence ratio (PR) with 95% confidence intervals (95%CI), multimorbidity presented associations with TV-viewing in general [from OR (95%CI) 1.10 (1.03–1.18) in 2 to < 3 h/d, to OR (95%CI) 1.57 (1.40–1.76) in ≥ 6 h/d], except in 2 to < 3 h/d time category for male and 35 to 49 years, and all time categories for 18 to 34 years. In addition, TV-viewing was associated with an increasing number of chronic conditions, all greater in ≥ 6 h/d [2 conditions - PR (95%CI) 1.24 (1.08–1.43); 3 conditions - PR (95%CI) 1.74 (1.45–2.08); 4 or more conditions - PR (95%CI) 2.29 (1.93–2.73)], except in 2 conditions on 2 to < 3 h/d. Other types of screen-based behaviors were only associated with multimorbidity among males [≥ 6 h/d: OR (95%CI) 1.22 (1.01–1.48)] and older individuals (65 years) in some time categories [3 to < 6 h/d: OR (95%CI) 1.98 (1.42–2.77) and ≥ 6 h/d: OR (95%CI) 1.73 (1.06–2.84)].

**Conclusion:**

Intervention strategies for reducing screen time in Brazilian adults should focus mainly on TV-viewing, which seems to be associated with more harmful conditions than time on other types of screen-based behaviors.

**Supplementary Information:**

The online version contains supplementary material available at 10.1186/s12889-022-14365-5.

## Introduction

Multimorbidity, a condition characterized by living with two or more chronic diseases, is a significant concern in public health [1]. The presence of multimorbidity increases mortality risk, and this risk increases according to the number of chronic diseases [2]. In addition, a considerable economic burden, mainly social costs, is associated with multimorbidity [3]. Currently, the overall pooled prevalence of multimorbidity is estimated at around 33% worldwide, ranging from 3.5 to 70% among high-income countries, and between 1% and 90% among low-middle income countries [4], with a higher prevalence among older adults.

There are several socioeconomic, clinical, and behavioral factors associated with multimorbidity, including sex, age, income, obesity, smoking, physical inactivity, and sedentary behavior [5–7]. Sedentary behavior has been investigated recently due to its associations with an increased risk of death from all causes and cardiovascular disease, an increased incidence of cardiovascular disease and type 2 diabetes, as well as being associated with the incidence of some types of cancer [8]. Furthermore, previous studies identified direct associations between multimorbidity and different types of sedentary behavior assessments, such as accelerometer-defined sedentary time [9, 10], reported sitting, [11, 12] and television time [13]. The variation between methods and metrics used to assess sedentary behavior in the previous papers imposes difficulties when comparing studies, and the reported associations with health outcomes may vary according to the study characteristics (i.e., domains, contexts, patterns) [14].

In Brazil, a previous study identified positive associations between TV-viewing and multimorbidity in adults and older adults living in state capitals [15]. However, there are no investigations into the other types of screen-based behaviors using a nationwide representative sample. Given that the time spent watching television by Brazilian adults has shown a downward trend [16] and the possibilities for screen-based behaviors have hugely expanded recently, it is crucial to investigate whether other types of screens show similar associations. Thus, our study is the first to investigate these associations in a nationally representative sample of Brazil and consider another type of screen-based behavior.

Therefore, we aimed to investigate the association between multimorbidity and two types of screen-based behaviors (TV-viewing and other screens) in Brazilian adults. Investigating these specific associations can foment interventions focusing on reducing sedentary behaviors more associated with adverse health outcomes.

## Methods

### Sample

We used data from the 2019 Brazilian National Health Survey. This survey is carried out by the Brazilian Ministry of Health in partnership with the Brazilian Institute of Geography and Statistics (IBGE – Portuguese acronym) and aims to characterize the health and lifestyle of the population. The survey included household-based interviews in a nationally representative sample of Brazilian inhabitants aged at least 15 years. The sample selection was cluster-based, with simple randomization, performed in three stages. The first stage aimed to select the primary sampling units (census tracts). The second stage aimed to select the households by simple randomization in each primary unit. Finally, the third stage consisted of a simple random sampling of residents at least 15 years old in each selected household. The Brazilian Council of Ethics in Research approved all procedures according to the Helsinki declaration. More details about the methods have been previously published elsewhere [17]. All variables were evaluated through self-report interviews. From the initial 100,541 selected households, 94,114 interviews were conducted. Due to missing data and after excluding individuals under 18 years of age (since the study’s target audience was adults), the final sample for this study was composed of 87,678 adults.

### Multimorbidity

Multimorbidity was defined as the diagnosis of two or more of the following chronic diseases, clinical conditions, or cardiometabolic risk factors: high blood pressure, diabetes, high cholesterol, heart disease, stroke, asthma/bronchitis, arthritis/rheumatism, chronic back pain, pulmonary disease, cancer, and kidney disease. These conditions were selected a priori among all chronic physical conditions with public health data available, based on the previous studies [11, 15, 18]. The questions used for each chronic condition were: *“Has a doctor ever given you the diagnosis of high blood pressure?”, “Has a doctor ever given you the diagnosis of diabetes”* and subsequently one question with the same structure for each one of the following clinical conditions: high cholesterol, heart disease, stroke, asthma or bronchitis, arthritis or rheumatism, chronic back pain, pulmonary disease, cancer, and kidney disease. The answers for each question were “yes” or “no.” Self-reported weight and height were used to calculate the body mass index, and obesity was defined using a cut-off of 30 kg/m^2^[19].

### Screen-based behaviors

The exposure variables were the types of screen-based behaviors, namely, TV-viewing and other screens (computer, tablet, or cell phone, excluding TV). TV-viewing was assessed by the question: *“On average, how many hours per day do you usually watch television?”*. The time spent on other screens was assessed by the question *“In one day, how many hours of your free time (excluding work), do you usually use a computer, tablet, or cell phone for leisure, such as: using social networks, to watch the news, videos, play, etc.?”*. The possible answers were: < 1 h/day, 1 to < 2 h/day, 2 to < 3 h/day, 3 to < 6 h/day, ≥ 6 h/day, and I don’t watch television or use a computer, tablet, or cell phone. Considering previous studies that used the < 2 h/day cut-off point to investigate associations between television time and chronic diseases in Brazilian adults [15, 20], we considered the first two response options and non-use in the same category, and thus, the responses were categorized as < 2 h/day, 2 to < 3 h/day, 3 to < 6 h/day, and ≥ 6 h/day. The shortest time category was the reference category for analyses.

### Confounders

The covariates were chosen according to the previous studies [6, 21], including sex (male or female), age group (18 to 34 years, 35 to 49 years, 50 to 64 years, or ≥ 65 years), ethnicity (white, black, mixed, other, or not informed), schooling (no formal instruction, less than secondary, secondary, or more than secondary), current tobacco smoking (yes or no), alcohol consumption (none or less than 1 day/month = no; once or more a month = yes), soft drink consumption (none or less than 1 day/week = no; 1 to 7 days/week = yes), fruit consumption (none or less than 5 days/week = no; 5 or more days/week = yes), and leisure physical activity (< 150 min/week or ≥ 150 min/week).

### Statistical analysis

Percentages and 95% confidence intervals were used to describe the prevalence of each outcome and to compare the groups. Crude and adjusted binary logistic regression models were created to analyze the association between multimorbidity and time categories of the different types of screen-based behaviors, stratified by sex and age group. Two sensitivity analyses were conducted. In the first, we built crude and adjusted multinomial logistic regression models to analyze the association between the number of chronic conditions and screen-based behaviors, classifying them as 0 or 1 (reference), 2, 3, or 4+ [21], to assess the intensity of multimorbidity. In the second sensitivity analysis, we analyzed the association between each condition and screen-based behavior using crude and adjusted logistic regression models. Linear regression models were also used to estimate the Variance Inflation Factor (VIF), whose results of 1.11 (TV-viewing) and 1.15 (other screens), indicating a lack of multicollinearity in the adjusted models of the main analyses. All analyses included sample weight and were performed using Stata software, version 15.0.

## Results

Table 1 shows the sample distribution according to the proportion of multimorbidity. The prevalence of multimorbidity was higher among females, older people, lower schooling, those reporting no consumption of alcohol or tobacco, low consumption of soft drinks, high fruit consumption, and those practicing less than 150 min of physical activity per week. Regarding screen-based behavior, a higher prevalence of multimorbidity was observed among those who spend six or more hours per day on TV-viewing and less than two hours per day on other screens.

Percentage of TV-viewing and others screens according sex and age groups are presented in Supplementary Table 1. For both TV-viewing and other screens, the highest proportions are found in < 2 h/day category, being higher in men and among the youngest adults for TV-viewing and among men and older adults to other screens in this category of time. On the other hand, the highest proportions of those who spend more time (≥ 6 h/day) on TV-viewing are found in females and among the older adults, and for other screens, in males and among the youngest adults.

**Table 1:** Sample characteristics stratified by the presence of multimorbidity (n = 87,678)

		Multimorbidity
**Variable**	**Categories**	**Yes % (95%CI)**
**Sex**	Male	25.14 (24.40–25.89)
	Female	35.55 (34.79–36.31)
**Age groups**	18 to 34 years	9.53 (8.90–10.21)
	35 to 49 years	25.00 (24.04–26.00)
	50 to 64 years	46.17 (45.01–47.33)
	≥ 65 years	60.91 (59.67–62.14)
**Ethnicity**	White	32.36 (31.50–33.24)
	Black	31.54 (30.01–33.11)
	Mixed	28.59 (27.84–29.36)
	Other	32.75 (27.95–37.94)
	Not informed	6.48 (0.73–39.50)
**Schooling**	No formal instruction	46.89 (44.83–48.90)
	Less than secondary	36.39 (35.56–37.23)
	Secondary	22.44 (21.58–23.33)
	More than secondary	26.61 (25.36–27.89)
**Alcohol consumption**	None or less than 1 day/month	33.37 (32.73–34.02)
	Once or more a month	24.28 (23.34–25.24)
**Current tobacco smoking**	No	31.02 (30.45–31.60)
	Yes	27.85 (26.37–29.37)
**Soft drink consumption**	None or less than 1 day/week	36.39 (35.64–37.14)
	1 to 7 days/week	25.01 (24.26–25.78)
**Fruits consumption**	None or less than 5 days/week	26.57 (25.88–27.26)
	5 or more days/week	35.58 (34.75–36.41)
**Leisure PA**	< 150 min/week	32.49 (31.87–33.13)
	≥ 150 min/week	25.46 (24.45–26.50)
**TV-viewing**	< 2 h/day	27.57 (26.88–28.28)
	2 to < 3 h/day	31.86 (30.71–33.03)
	3 to < 6 h/day	35.34 (33.96–36.74)
	≥ 6 h/day	43.29 (40.95–45.66)
**Other screens**	< 2 h/day	35.91 (35.24–36.58)
	2 to < 3 h/day	23.30 (21.89–24.77)
	3 to < 6 h/day	20.49 (19.12–21.93)
	≥ 6 h/day	18.27 (16.76–19.88)

Note. %: percentage in the weighted sample. 95%CI: confidence interval in the weighted sample. PA: physical activity. TV: television

The associations between multimorbidity and screen-based behaviors in all individuals and stratified by sex are presented in Table 2. For adjusted models, considering both sexes there was an association between multimorbidity and all categories of TV-viewing, while no significant association was observed with any time category for other screens. When categorizing TV-viewing by sex, the associations occurred in the two highest categories for males and all-time categories for females. An association for other screens was found only among men in the highest time category.

**Table2:** Association between screen-based behaviors and multimorbidity for all participants and stratified by sex

		Multimorbidity		
		**OR (95%CI)**		
**Screen-based behaviors**		**All**	**Male**	**Female**
**Crude**				
**TV-viewing**	< 2 h/d	ref	ref	ref
	2 to < 3 h/d	**1.23** **(1.15–1.31)**	**1.13** **(1.02–1.24)**	**1.30** **(1.20–1.42)**
	3 to < 6 h/d	**1.44** **(1.34–1.54)**	**1.32** **(1.18–1.47)**	**1.51** **(1.38–1.66)**
	≥ 6 h/d	**2.01** **(1.81–2.22)**	**2.06** **(1.74–2.44)**	**1.89** **(1.66–2.15)**
**Other screens**	< 2 h/d	ref	ref	ref
	2 to < 3 h/d	**0.54** **(0.50–0.59)**	**0.61** **(0.53–0.70)**	**0.49** **(0.44–0.54)**
	3 to < 6 h/d	**0.46** **(0.42–0.50)**	**0.50** **(0.43–0.57)**	**0.42** **(0.38–0.48)**
	≥ 6 h/d	**0.40** **(0.36–0.44)**	**0.47** **(0.39–0.56)**	**0.35** **(0.30–0.40)**
**Adjusted**				
**TV-viewing**	< 2 h/d	ref	ref	ref
	2 to < 3 h/d	**1.10** **(1.03–1.18)**	1.03 (0.93–1.14)	**1.14** **(1.04–1.26)**
	3 to < 6 h/d	**1.22** **(1.13–1.32)**	**1.20** **(1.07–1.34)**	**1.21** **(1.09–1.34)**
	≥ 6 h/d	**1.57** **(1.40–1.76)**	**1.88** **(1.57–2.27)**	**1.36** **(1.18–1.56)**
**Other screens**	< 2 h/d	ref	ref	ref
	2 to < 3 h/d	1.03 (0.94–1.13)	1.10 (0.94–1.27)	0.99 (0.88–1.11)
	3 to < 6 h/d	1.05 (0.95–1.15)	1.08 (0.92–1.25)	1.03 (0.91–1.17)
	≥ 6 h/d	1.09 (0.96–1.23)	**1.22** **(1.01–1.48)**	1.00 (0.86–1.16)

Note. Logistic regression analyses adjusted by sex (except for male and female), age groups, ethnicity, schooling, alcohol consumption, current tobacco smoking, soft drink consumption, fruit consumption, and leisure physical activity. TV: television. h/d: hours per day. ref: reference category. OR: odds ratio. 95%CI: 95% confidence interval. OR values with a significance level < 0.05 are highlighted in bold

Table 3 presents associations stratified by age groups. For adjusted models, about TV-viewing, associations were found in the two highest time categories in the age group between 35 and 49 years, and in all time categories in the age groups between 50 and 64 years and from 65 years, while for other screens, associations were found in the two highest time categories only among those from 65 years old.

**Table 3:** Association between screen-based behaviors and multimorbidity stratified by age group categories

		Multimorbidity			
		**OR (95%CI)**			
**Screen-based behaviors**		**18 to 34 y**	**35 to 49 y**	**50 to 64 y**	**≥ 65 y**
**Crude**					
**TV-viewing**	< 2 h/d	ref	ref	ref	ref
	2 to < 3 h/d	0.94 (0.77–1.15)	1.02 (0.89–1.17)	**1.18** **(1.05–1.32)**	**1.20** **(1.06–1.37)**
	3 to < 6 h/d	1.13 (0.92–1.39)	**1.20** **(1.02–1.41)**	**1.24** **(1.10–1.41)**	**1.38** **(1.20–1.59)**
	≥ 6 h/d	1.38 (0.99–1.94)	**1.61** **(1.27–2.04)**	**1.84** **(1.52–2.22)**	**1.73** **(1.43–2.09)**
**Other screens**	< 2 h/d	ref	ref	ref	ref
	2 to < 3 h/d	0.88 (0.71–1.10)	1.05 (0.90–1.22)	1.00 (0.85–1.17)	1.15 (0.87–1.52)
	3 to < 6 h/d	0.97 (0.80–1.18)	0.98 (0.82–1.17)	1.14 (0.96–1.36)	**1.81** **(1.28–2.54)**
	≥ 6 h/d	1.17 (0.95–1.45)	0.96 (0.80–1.16)	1.15 (0.89–1.48)	1.54 (0.94–2.50)
**Adjusted**					
**TV-viewing**	< 2 h/d	ref	ref	ref	ref
	2 to < 3 h/d	0.94 (0.77–1.15)	1.03 (0.90–1.17)	**1.17** **(1.05–1.32)**	**1.20** **(1.05–1.37)**
	3 to < 6 h/d	1.14 (0.93–1.41)	**1.20** **(1.02–1.40)**	**1.22** **(1.08–1.39)**	**1.36** **(1.18–1.56)**
	≥ 6 h/d	1.38 (0.98–1.93)	**1.54** **(1.21–1.96)**	**1.77** **(1.46–2.16)**	**1.63** **(1.34–1.98)**
**Other screens**	< 2 h/d	ref	ref	ref	ref
	2 to < 3 h/d	0.90 (0.73–1.12)	1.10 (0.94–1.29)	1.05 (0.89–1.24)	1.18 (0.88–1.58)
	3 to < 6 h/d	1.00 (0.81–1.22)	1.03 (0.86–1.22)	1.16 (0.97–1.38)	**1.98** **(1.42–2.77)**
	≥ 6 h/d	1.20 (0.96–1.50)	1.02 (0.84–1.23)	1.16 (0.89–1.52)	**1.73** **(1.06–2.84)**

Note. Logistic regression analyses adjusted by sex, ethnicity, schooling, alcohol consumption, current tobacco smoking, soft drink consumption, fruits consumption, and leisure physical activity. TV: television. h/d: hours per day. y: years. ref: reference category. OR: odds ratio. 95%CI: 95% confidence interval. OR values with a significance level < 0.05 are highlighted in bold

Associations between the number of chronic conditions and screen-based behaviors are shown in Table 4. For adjusted models, considering TV-viewing, associations increased according to the number of chronic conditions. No associations were found for other screens in any time exposure categories.

**Table4:** Association between screen-based behaviors and the number of chronic conditions

		Number of chronic conditions PR (95%CI)			
**Screen-based behaviors**		**0 or 1**	**2**	**3**	**4 or more**
**Crude**					
TV-viewing	< 2 h/d	ref	ref	ref	ref
	2 to < 3 h/d	ref	**1.15 (1.06–1.24)**	**1.31 (1.17–1.46)**	**1.35 (1.19–1.52**)
	3 to < 6 h/d	ref	**1.28 (1.17–1.40)**	**1.51 (1.34–1.71)**	**1.75 (1.55–1.98)**
	≥ 6 h/d	ref	**1.44 (1.27–1.65)**	**2.24 (1.89–2.65)**	**3.29 (2.75–3.75)**
Other screens	< 2 h/d	ref	ref	ref	ref
	2 to < 3 h/d	ref	**0.64 (0.57–0.70)**	**0.52 (0.44–0.62)**	**0.37 (0.32–0.44)**
	3 to < 6 h/d	ref	**0.55 (0.50–0.62)**	**0.38 (0.32–0.46)**	**0.35 (0.29–0.42)**
	≥ 6 h/d	ref	**0.51 (0.44–0.57)**	**0.32 (0.26–0.39)**	**0.26 (0.20–0.33)**
**Adjusted**					
TV-viewing	< 2 h/d	ref	ref	ref	ref
	2 to < 3 h/d	ref	1.05 (0.97–1.14)	**1.16 (1.04–1.30)**	**1.18 (1.03–1.34)**
	3 to < 6 h/d	ref	**1.14 (1.04–1.24)**	**1.27 (1.11–1.44)**	**1.39 (1.22–1.58)**
	≥ 6 h/d	ref	**1.24 (1.08–1.43)**	**1.74 (1.45–2.08)**	**2.29 (1.93–2.73)**
Other screens	< 2 h/d	ref	ref	ref	ref
	2 to < 3 h/d	ref	1.02 (0.91–1.13)	1.09 (0.90–1.31)	0.97 (0.81–1.17)
	3 to < 6 h/d	ref	1.01 (0.90–1.14)	0.99 (0.81–1.20)	1.19 (0.97–1.45)
	≥ 6 h/d	ref	1.06 (0.92–1.23)	1.02 (0.83–1.25)	1.13 (0.88–1.46)

Note. Multinomial regression analyses adjusted by sex, age groups, ethnicity, schooling, alcohol consumption, current tobacco smoking, soft drink consumption, fruits consumption, and leisure physical activity. TV: television. h/d: hours per day. ref: reference category. PR: prevalence ratio. 95%CI: 95% confidence interval. PR values with a significance level < 0.05 are highlighted in bold

The Supplementary Table 2 (see Supplementary Material 2) presents the data for associations between each chronic condition and screen-based behaviors. Considering adjusted models, for TV-viewing, associations with high blood pressure were found in all time categories; associations with diabetes, heart disease, stroke, and obesity in the two highest time categories; associations with asthma/bronchitis and pulmonary disease only in the ≥ 6 h/day category; associations with arthritis/rheumatism only in the lowest time category; and associations with high cholesterol in the lowest and highest time categories. For other screens, associations were found in all time categories for asthma/bronchitis and chronic back pain. However, for the latter, the associations were positive; associations with pulmonary disease and obesity were found in the 3 to < 6 h/day category; and associations with heart disease, kidney disease, and arthritis/rheumatism were found only in the ≥ 6 h/day category, however, the association with this latter condition was also positive.

## Discussion

We found significant associations between TV-viewing and multimorbidity in this nationally representative sample of almost 88,000 Brazilian adults of both sexes. Importantly, we also observed an apparent dose-response relationship, since the higher the TV-viewing time category, the higher the associations. On the other hand, in general, we found no association between other screen use and multimorbidity, except among men (highest time category) and older individuals (two highest time categories). Sensitivity analyses revealed that the association with TV-viewing time also increased with the increasing number of chronic conditions, as well as that among those with ≥ 3 h/day of TV-viewing, there were increased odds of multimorbidity in all age-groups from 35 years and older.

Our findings confirm previous studies that found associations between multimorbidity and TV-viewing in both sexes. In European adults, associations were found in all time categories for women and men, from at least 2 h/day of exposure [13], compared to 3 h/day in our study. Previous studies confirm greater associations with multimorbidity in women than men [5, 22, 23]. These differences can be explained by the fact that women have a longer life expectancy and spend more years with multimorbidity [23] as well as those women seek health services more frequently than men [24]. Our results showed that 35.5% of women had the outcome of multimorbidity, while the proportion among men was lower (25.1%). However, the associations were similar in both sexes, with a slight predominance among women, with a significant association in all time categories evaluated. In any case, our data reinforce that TV-viewing is associated with highly prevalent adverse health outcomes [25–27].

The analyses stratified by age group confirmed these associations between multimorbidity and TV-viewing, except among the youngest age group. A previous study with a sample of Brazilians identified associations with ≥ 2 h of TV-viewing, both among adults and older adults [15]. However, that study only used four chronic conditions to classify multimorbidity, while we considered 12 chronic conditions; the authors used a nationally representative sample of state capitals while we used state capitals and non-capitals, and they investigated associations with two different time categories while we investigated four. In addition, due to the cut-off point used in this study to categorize age groups, it was impossible to identify associations with younger adults to compare our findings. There is evidence that age correlates with multimorbidity and that the prevalence among older adults is higher than other age categories [7, 22, 28, 29]. In our study, the proportion of individuals with multimorbidity was only 9.5% among younger adults, and 60.9% among older adults, which could explain these associations. Although it is impossible to establish causality, this information ratifies the importance of reducing sedentary behaviors, as expressed by screen time, mainly TV-viewing, with particular attention to older adults [30].

In addition to multimorbidity, we identified that TV-viewing was also associated with a higher number of chronic conditions. A previous study with a sample of Brazilians living in state capitals has already shown associations with ≥ 2 h/day of TV-viewing and multimorbidity, with higher odds observed with an increasing number of chronic conditions [15]. A review study with data from six low-middle-income countries demonstrated that the prevalence of sedentary behavior increased with the number of chronic conditions (from zero to four), however, the measure used was sitting time, and the cut-off point was from 8 h/day [12]. Another study, which also used sitting time, found that sedentary behavior increased with the number of chronic conditions, and people with four or more conditions were significantly more likely to present elevated sedentary behavior [11]. From these results and the results found in our study, it appears that the number of chronic conditions influences the prevalence ratio values of these associations, which seems to be a dose-response trend, but which can only be confirmed in a longitudinal study to demonstrate the cause-and-effect relationship.

Concerning time on other screens, the associations were only identified in stratified analyses, in the highest time category for males and the highest two time categories for the age group from 65 years. In our study, the proportion of individuals who spent more time on other screens was higher among those who did not have multimorbidity, and perhaps this contributed to the associations being found only among these subgroups, which is also the age group that proportionally presents more multimorbidity compared to other age groups. In addition, age possibly has some moderating effect on this association because when the analysis was stratified by age, the association was found for the group over 65 years old.

Despite investigating isolated cardiometabolic markers, instead of multimorbidity, a previous study with an Asian urban population also found associations with TV-viewing but not with other screens (computer use and reading time). The authors believe that dietary factors and the body mass index are potential moderators that explain these associations [31], mainly because of poor habits associated with TV-viewing [26], which has not yet been confirmed for other screens. Another aspect that may explain the differences in results between TV-viewing and other screens is the time between the beginning of exposure and the appearance of the outcome, and longitudinal studies are needed to verify whether the effects of time on other screens will be observed among younger people in the future. In addition, TV-viewing and other screens have different correlates about age and socioeconomic variables. While higher TV-viewing is associated with lower educational status, other screens are associated with younger adults [32]. Also, the two behaviors may present different characteristics concerning, for example, the number of bouts, breaks, and the overlapping of behaviors, such as standing while using the cell phone.

Although the trend toward decreasing television time among adult Brazilians has already been observed [16], we did not identify this migration to other screens in our study. In addition to the fact that the prevalence of individuals with multimorbidity is higher among those who spend more time on TV-viewing, the lack of association between multimorbidity and other types of screen-based behaviors (except those that appear in the stratifications by sex and age) indicate that the two types of behaviors have different relationships with the evaluated outcome. Despite the reduction of TV-viewing in recent years, the percentage of people with 2 h/day or more of TV-viewing is still considerable (42.7% - see Supplementary Table 1 in Supplementary Material 1). Thus, it becomes important to observe both the behavior that is more typical and the behavior that is becoming more and more frequent.

Concerning strengths, our study was the first in Brazil with a representative national sample to use two measures of sedentary behavior (TV-viewing and other types of screen) to investigate associations with multimorbidity, stratified by sex, age group, and according to the number of chronic conditions. Our findings elucidate differences in associations between types of screen-based behaviors and health outcomes. This may help to direct specific actions for the control and monitoring of this condition.

Our study has some limitations. As this is a cross-sectional study, it is impossible to infer cause and effect. Furthermore, other screens (computer, tablet, or cell phone) were evaluated together, which made it impossible to analyze associations for each type of screen-based behavior. In addition, all variables were self-reported, and the choice of chronic conditions that constituted the multimorbidity variable could make the associations specific to the conditions adopted in this study. However, epidemiological studies carried out in low-middle income countries used an average of eight to 12 chronic conditions to compose a measure of multimorbidity, and our study was based on a list of 25 conditions most widely considered to compose the measure in these studies [33]. It is also essential to consider the possibility of reverse causality, since people with multimorbidity may present increased body mobility limitations, which might predispose them to higher screen-based behaviors.

## Conclusion

We concluded that multimorbidity presented associations with TV-viewing. These associations were present in both sexes, and all age group categories, except among the youngest adults. In addition, TV-viewing is also associated with an increasing number of chronic conditions. On the other hand, other types of screen-based behaviors are only associated with multimorbidity in some time categories among males and older adults. Therefore, intervention strategies for reducing screen time in Brazilian adults should focus primarily on TV-viewing, which seems to be associated with more deleterious conditions than time spent on other types of screen-based behaviors.

## Electronic supplementary material

Below is the link to the electronic supplementary material.

Supplementary Material 1

Supplementary Material 2

## Data Availability

Data from the National Health Survey is available on the Brazilian Institute of Geography and Statistics website (https://www.ibge.gov.br/estatisticas/sociais/saude/9160-pesquisa-nacional-de-saude.html?=&t=downloads).
