# Supplementary material for: Multimorbidity is associated with TV-viewing, but not with other types of screen-based behaviors in Brazilian adults
Source: BMC Public Health. 2022 Oct 31;22:1991. doi: 10.1186/s12889-022-14365-5 (PMC9623956; doi:10.1186/s12889-022-14365-5)
Supplement: Supplementary file 1 — Supplementary Material 1 [file 12889_2022_14365_MOESM1_ESM.docx]

Supplementary Table 1 **–** Proportion of TV-viewing and other screens stratified by sex and age groups (n = 87,678).

|  | | **Percentage % (95%CI)** | | | | | | |
| --- | --- | --- | --- | --- | --- | --- | --- | --- |
|  | |  | **Sex** | | **Age groups** | | | |
| **Screen-based behaviors** | | **All** | **Male** | **Female** | **18 to 34 y** | **35 to 49 y** | **50 to 64 y** | **≥ 65 y** |
| **TV-viewing** | < 2 h/d | 57.3  (56.7-57.9) | 58.5  (57.6-59.3) | 56.2  (55.4-57.0) | 61.4  (60.2-62.5) | 60.3  (59.2-61.4) | 54.7  (53.6-55.9) | 46.8  (45.5-48.1) |
|  | 2 to < 3 h/d | 21.0  (20.5-21.5) | 21.0  (20.3-21.7) | 21.0  (20.4-21.7) | 18.5  (17.7-19.4) | 21.9  (21.0-22.8) | 22.8  (21.9-23.8) | 21.6  (20.6-22.7) |
|  | 3 to < 6 h/d | 15.9  (15.4-16.3) | 15.5  (14.8-16.1) | 16.2  (15.7-16.8) | 14.9  (14.0-15.7) | 13.7  (12.9-14.5) | 16.3  (15.6-17.2) | 21.5  (20.5-22.7) |
|  | ≥ 6 h/d | 5.8  (5.6-6.1) | 5.0  (4.7-5.4) | 6.5  (6.1-6.9) | 5.2  (4.7-5.8) | 4.1  (3.7-4.5) | 6.1  (5.6-6.7) | 10.0  (9.3-10.8) |
| **Other screens** | < 2 h/d | 64.5  (63.9-65.1) | 65.0  (64.1-65.8) | 64.1  (63.3-64.9) | 37.8  (36.7-38.9) | 66.2  (65.2-67.2) | 80.8  (79.9-81.7) | 91.2  (90.3-91.2) |
|  | 2 to < 3 h/d | 13.4  (13.0-13.8) | 13.1  (12.5-13.8) | 13.7  (13.1-14.2) | 19.1  (18.2-20.1) | 15.2  (14.4-16.0) | 9.5  (8.8-10.2) | 4.3  (3.8-4.9) |
|  | 3 to < 6 h/d | 13.5  (13.1-14.0) | 13.3  (12.7-14.0) | 13.7  (13.1-14.3) | 24.5  (23.4-25.6) | 12.6  (11.9-13.4) | 6.8  (6.2-7.3) | 3.1  (2.7-3.7) |
|  | ≥ 6 h/d | 8.5  (8.2-8.9) | 8.6  (8.1-9.1) | 8.5  (8.0-9.0) | 18.6  (17.7-19.5) | 6.0  (5.6-6.5) | 2.9  (2.6-3.3) | 1.3  (1.1-1.7) |

Note. %: percentage in the weighted sample. 95%CI: confidence interval in the weighted sample. TV: television. h/d: hours per day. y: years.
