# Supplementary material for: Multimorbidity is associated with TV-viewing, but not with other types of screen-based behaviors in Brazilian adults
Source: BMC Public Health. 2022 Oct 31;22:1991. doi: 10.1186/s12889-022-14365-5 (PMC9623956; doi:10.1186/s12889-022-14365-5)
Supplement: Supplementary file 2 — Supplementary Material 2 [file 12889_2022_14365_MOESM2_ESM.docx]

Supplementary Table 2 **-** Association between screen-based behaviors and chronic conditions.

|  |  | **TV-viewing** | | | | **Other screens** | | | |
| --- | --- | --- | --- | --- | --- | --- | --- | --- | --- |
| **Chronic conditions** |  | **< 2 h/d** | **2 to < 3 h/d** | **3 to < 6 h/d** | **≥ 6 h/d** | **< 2 h/d** | **2 to < 3 h/d** | **3 to < 6 h/d** | **≥ 6 h/d** |
| **High blood pressure** | Crude OR (95%CI) | ref | **1.25 (1.17-1.34)** | **1.47 (1.36-1.58)** | **2.00 (1.80-2.23)** | ref | **0.44 (0.40-0.49)** | **0.36 (0.32-0.40)** | **0.30 (0.26-0.34)** |
|  | Adjusted OR (95%CI) | ref | **1.11 (1.03-1.19)** | **1.20 (1.11-1.31)** | **1.48 (1.31-1.67)** | ref | 0.99 (0.88-1.11) | 1.00 (0.89-1.12) | 1.02 (0.88-1.18) |
| **Diabetes** | Crude OR (95%CI) | ref | **1.22 (1.09-1.36)** | **1.55 (1.39-1.73)** | **2.49 (2.16-2.86)** | ref | **0.41 (0.35-0.47)** | **0.33 (0.28-0.40)** | **0.31 (0.24-0.38)** |
|  | Adjusted OR (95%CI) | ref | 1.09 (0.97-1.22) | **1.26 (1.12-1.42)** | **1.80 (1.54-2.11)** | ref | 0.92 (0.78-1.09) | 0.93 (0.77-1.13) | 1.06 (0.84-1.34) |
| **High Cholesterol** | Crude OR (95%CI) | ref | **1.23 (1.13-1.34)** | **1.25 (1.15-1.36)** | **1.45 (1.28-1.65)** | ref | **0.63 (0.57-0.70)** | **0.53 (0.48-0.60)** | **0.50 (0.42-0.58)** |
|  | Adjusted OR (95%CI) | ref | **1.14 (1.04-1.24)** | 1.07 (0.98-1.17) | **1.15 (1.01-1.31)** | ref | 0.96 (0.86-1.08) | 0.93 (0.83-1.05) | 1.02 (0.86-1.20) |
| **Heart disease** | Crude OR (95%CI) | ref | **1.23 (1.08-1.40)** | **1.55 (1.35-1.79)** | **2.41 (2.03-2.88)** | ref | **0.58 (0.48-0.70)** | **0.52 (0.41-0.66)** | **0.50 (0.40-0.63)** |
|  | Adjusted OR (95%CI) | ref | 1.12 (0.98-1.28) | **1.32 (1.13-1.54)** | **1.82 (1.51-2.19)** | ref | 1.16 (0.95-1.41) | 1.27 (0.99-1.62) | **1.46 (1.15-1.86)** |
| **Stroke** | Crude OR (95%CI) | ref | **1.32 (1.05-1.66)** | **1.70 (1.41-2.06)** | **3.61 (2.86-4.55)** | ref | **0.28 (0.20-0.37)** | **0.31 (0.19-0.51)** | **0.19 (0.12-0.29)** |
|  | Adjusted OR (95%CI) | ref | 1.21 (0.96-1.53) | **1.41 (1.16-1.72)** | **2.55 (2.01-3.23)** | ref | 0.74 (0.54-1.02) | 1.03 (0.63-1.68) | 0.76 (0.49-1.18) |
| **Asthma/**  **bronchitis** | Crude OR (95%CI) | ref | 0.97 (0.84-1.13) | 0.89 (0.77-1.03) | **1.41 (1.12-1.78)** | ref | **1.44 (1.23-1.69)** | **1.44 (1.23-1.68)** | **1.64 (1.35-2.00)** |
|  | Adjusted OR (95%CI) | ref | 0.99 (0.85-1.14) | 0.91 (0.79-1.06) | **1.47 (1.16-1.87)** | ref | **1.31 (1.11-1.54)** | **1.30 (1.10-1.52)** | **1.48 (1.21-1.81)** |
| **Arthritis/**  **rheumatism** | Crude OR (95%CI) | ref | **1.30 (1.16-1.46)** | **1.39 (1.24-1.57)** | **1.75 (1.49-2.05)** | ref | **0.45 (0.39-0.53)** | **0.40 (0.33-0.47)** | **0.29 (0.24-0.36)** |
|  | Adjusted OR (95%CI) | ref | **1.15 (1.02-1.29)** | 1.09 (0.96-1.23) | 1.14 (0.96-1.36) | ref | 0.86 (0.73-1.01) | 0.91 (0.75-1.09) | **0.80 (0.64-1.00)** |
| **Chronic back pain** | Crude OR (95%CI) | ref | 1.06 (0.99-1.14) | **1.12 (1.03-1.21)** | **1.24 (1.10-1.39)** | ref | **0.64 (0.58-0.70)** | **0.56 (0.50-0.62)** | **0.48 (0.42-0.54)** |
|  | Adjusted OR (95%CI) | ref | 0.99 (0.92-1.07) | 1.00 (0.91-1.09) | 1.00 (0.89-1.13) | ref | **0.89 (0.80-0.98)** | **0.83 (0.74-0.93)** | **0.75 (0.66-0.86)** |
| **Pulmonary disease** | Crude OR (95%CI) | ref | 1.10 (0.81-1.48) | 1.19 (0.93-1.52) | **2.52 (1.85-3.44)** | ref | 0.80 (0.58-1.11) | 0.96 (0.70-1.32) | 0.85 (0.57-1.26) |
|  | Adjusted OR (95%CI) | ref | 1.04 (0.77-1.40) | 1.08 (0.84-1.39) | **2.13 (1.55-2.92)** | ref | 1.16 (0.84-1.61) | **1.50 (1.07-2.12)** | 1.42 (0.93-2.16**)** |
| **Cancer** | Crude OR (95%CI) | ref | 1.11 (0.93-1.33) | **1.43 (1.19-1.72)** | **1.70 (1.32-2.20)** | ref | **0.58 (0.46-0.72)** | **0.57 (0.43-0.75)** | **0.30 (0.21-0.43)** |
|  | Adjusted OR (95%CI) | ref | 0.95 (0.79-1.14) | 1.09 (0.91-1.32) | 1.15 (0.88-1.50) | ref | 0.97 (0.76-1.24) | 1.20 (0.90-1.62) | 0.79 (0.54-1.15) |
| **Kidney disease** | Crude OR (95%CI) | ref | 1.01 (0.80-1.28) | 1.17 (0.92-1.50) | **1.59 (1.14-2.22)** | ref | **0.62 (0.43-0.87)** | **0.72 (0.52-0.99)** | 0.96 (0.65-1.41) |
|  | Adjusted OR (95%CI) | ref | 0.96 (0.76-1.21) | 1.07 (0.83-1.38) | 1.31 (0.93-1.84) | ref | 1.02 (0.73-1.43) | 1.34 (0.95-1.89) | **1.97 (1.31-2.96)** |
| **Obesity** | Crude OR (95%CI) | ref | **1.10 (1.02-1.19)** | **1.27 (1.17-1.38)** | **1.53 (1.36-1.71)** | ref | 0.93 (0.85-1.02) | 1.04 (0.94-1.16) | 0.96 (0.86-1.07) |
|  | Adjusted OR (95%CI) | ref | 1.07 (1.00-1.15) | **1.24 (1.14-1.35)** | **1.46 (1.30-1.63)** | ref | 1.01 (0.91-1.11) | **1.16 (1.04-1.29)** | 1.09 (0.97-1.23) |

Note. Logistic regression analyses adjusted by sex, age group, ethnicity, schooling, alcohol consumption, current tobacco smoking, soft drink consumption, fruit consumption, and leisure physical activity. TV: television. h/d: hours per day. ref: reference category. OR: odds ratio. 95%CI: 95% confidence interval. OR values with a significance level < 0.05 are highlighted in bold.
